# Retraction: Temperature-regulated polymerization and swelling/collapsing/flocculation properties of hybrid nanospheres with magnetic cores and thermo/pH-sensitive nanogel shells

**DOI:** 10.1039/d0ra90104k

**Published:** 2020-10-19

**Authors:** Laura Fisher

**Affiliations:** Royal Society of Chemistry, Thomas Graham House, Science Park Milton Road Cambridge CB4 0WF UK advances-rsc@rsc.org

## Abstract

Retraction of ‘Temperature-regulated polymerization and swelling/collapsing/flocculation properties of hybrid nanospheres with magnetic cores and thermo/pH-sensitive nanogel shells’ by Rijun Gui *et al.*, *RSC Adv.*, 2014, **4**, 2797–2806, DOI: 10.1039/C3RA43919D.

The Royal Society of Chemistry hereby wholly retracts this *RSC Advances* article due to concerns with the reliability of the data in the published article.

The TEM image in Fig. 2a duplicates data in another publication, but reported as different materials.^1^

The TEM image in Fig. 2c duplicates data in another publication, but reported as a different material.^2^

Given the number and significance of the concerns about the validity of the data, the findings presented in this paper are no longer reliable.

Rijun Gui opposes this retraction. Hui Jin was contacted but did not respond.

Signed: Laura Fisher, Executive Editor, *RSC Advances*

Date: 21^st^ September 2020

## Supplementary Material
